# Metabolomics reveals the effect of sand roasting, freeze-drying, and different hydrothermal treatments on the physical, functional and bioactive profile of Rice bean (*Vigna umbellata*)

**DOI:** 10.1016/j.fochx.2026.104553

**Published:** 2026-10-07

**Authors:** Somaina Basumatary, Manish Kumar Singh, Baby Zaithanpuii Hmar, Arvind Kumar, Jagan Karthik Sugumar, Ankur Aggarwal, Himanshu Mishra, Kumari Amita Singh, Satyender Singh Yadav, Sunil Kumar, Vishal Kumar

**Affiliations:** a Department of Food Technology, School of Engineering and Technology, Mizoram University, Aizawl, Mizoram, India; b Department of Dairy Science and Food Technology, Institute of Agricultural Sciences, Banaras Hindu University, Varanasi, Uttar Pradesh, India; c Department of Food Technology, Faculty of Engineering, Karpagam Academy of Higher Education, Coimbatore, Tamil Nadu, India; d Department of Biochemistry, Institute of Science, Banaras Hindu University, Varanasi, Uttar Pradesh, India

**Keywords:** Rice bean, Bioactivity, Germination, Engineering properties, Freeze-drying, Sand roasting, Metabolomics, Antioxidant activity

## Abstract

This study comprehensively investigated the effects of five processing treatments, namely soaking (SK), germination (GR), sand roasting (SR), freeze-drying (FD), and pressure cooking (PC) on the physicochemical, functional, structural, antioxidant, and metabolomic characteristics of rice bean (*Vigna umbellata*), an underutilised legume with high nutritional potential. Significant variations (*p* ≤ 0.05) were observed among treatments. GR proved most effective in enhancing nutritional and bioactive quality, yielding the highest protein content (24.3%), total phenolics (8.42 mg GAE/g), flavonoids (4.22 mg QE/g), DPPH radical scavenging activity (72.67%), foaming capacity (32.82%), and emulsion stability (76.3%). PC maximised sphericity (68.85%), swelling index (5.31 g/g), and hydration properties, while SR enhanced oil absorption capacity (2.42 g/g). Untargeted HR-MS metabolomics detected 10,533 metabolite features, with GR samples showing the richest profile and enrichment of quercetin, kaempferol, and naringenin derivatives; 283 metabolites constituted the shared core metabolome. FT-IR revealed treatment-induced modifications in protein, lipid, and carbohydrate structures, and XRD confirmed A-type starch crystallinity, largely preserved by FD but disrupted by PC. These findings demonstrate that processing-specific structural modifications govern functionality, highlighting the targeted use of rice bean flour in functional food applications.

## Highlights

• Effects of five processing namely SK, PC, GR, SR and FD were studied on rice bean.
• The GR sample significantly enhanced functionality and shown rich metabolite profile.
• FT-IR reveals alterations in biomolecules, indicating the optimal processing conditions for rice beans.
• XRD confirmed A-type starch crystallinity, high in FD and SK but low in PC rice bean.
• Kaempferol, quercetin, naringenin derivatives, catechin, and organic acids were seen.

## Graphical abstract

**Figure:**
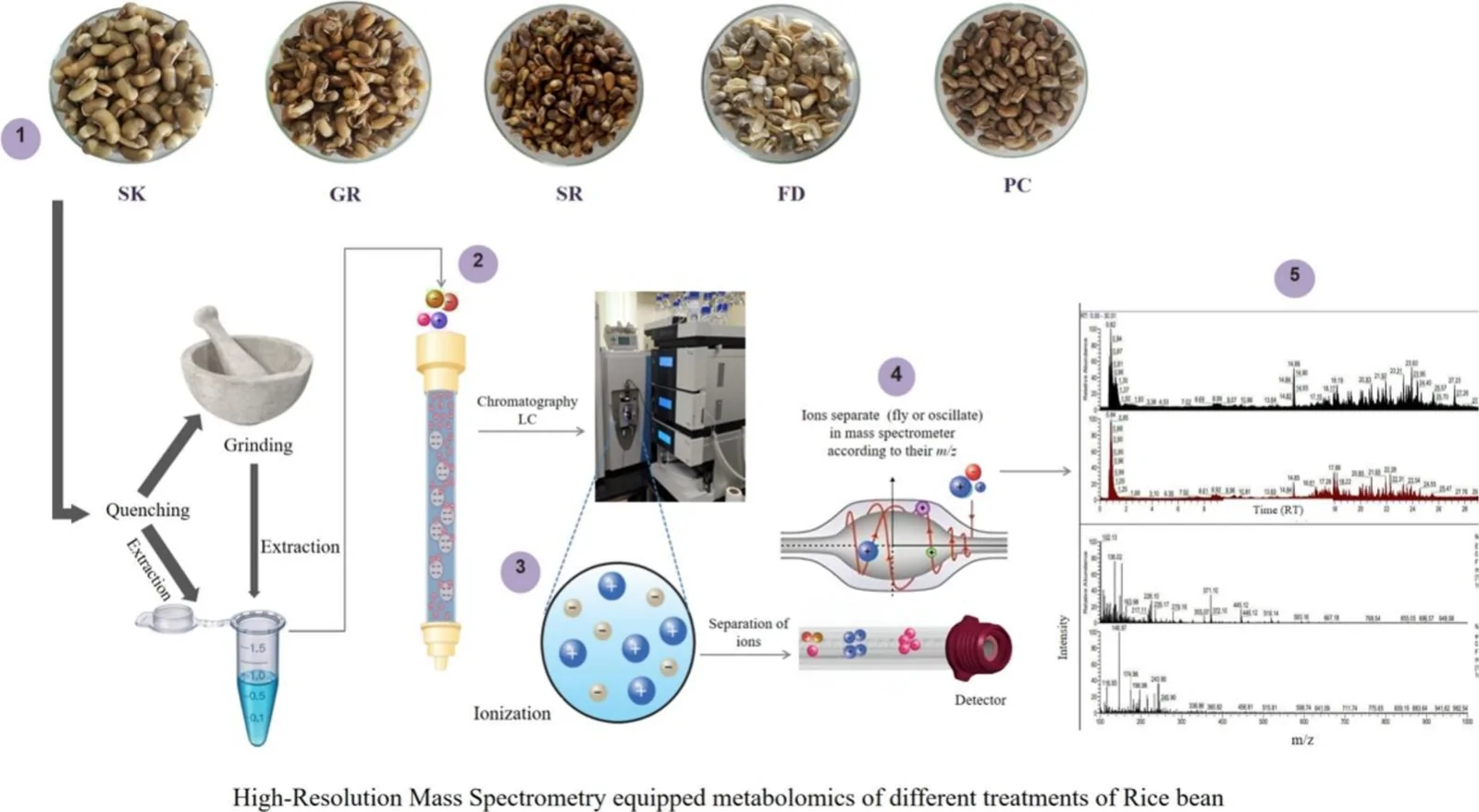

## 1 Introduction

Legumes are a primary source of plant-based alternative protein, particularly in developing regions across Africa, Asia, and South America (Lisciani et al., 2024). Staple legumes, i.e., soybean, pea, chickpea, lentil, and cowpea are integral to global food systems because of their high nutrient density, including protein (20–26%), dietary fibre, essential micronutrients, and lower carbohydrate content compared to cereals (Medendorp et al., 2022). Pulses also provide significant mineral content, including iron, zinc, calcium, magnesium, and potassium, which are critical for the nutritional security of low-income populations (Dissanayaka et al., 2021). Beyond macronutrients, legumes are rich in bioactive compounds (BACs), including phenolics, flavonoids, and alkaloids, which confer antioxidant, anti-inflammatory, and anti-diabetic properties (Lovegrove et al., 2023; Vijayakumar & M, 2021). Specific phytochemicals (ferulic acid, rutin, tannins, and saponins) exhibit anti-obesity, anti-cancer, and antibacterial activities (Chen et al., 2025; Yao et al., 2012). Rice bean extracts, for example, have demonstrated anti-inflammatory and anti-diabetic potential via α-glucosidase inhibition and cyclooxygenase-2 suppression (Sritongtae et al., 2017; Yao et al., 2012).

Rice bean (*Vigna umbellata L*.) is a warm-season underutilised legume belonging to the Fabaceae family and is widely cultivated in Southeast Asia in mixed cropping systems with maize, sorghum, and millet (Sritongtae et al., 2017). The seeds of the bean are either red or brown in colour, and the shape ranges from oval to cylindrical, with a size of ∼3 to 5 mm (Wadikar & Bepary, 2020). This bean has a good nutritional score, with protein (18–25%), complex carbohydrates including dietary fibre (55–65%), lipid (1–3%), B-complex vitamins, and minerals including calcium, magnesium, and iron (Wadikar & Bepary, 2020). Since the protein of rice bean contains a good amount of *Trp* and *Lys* amino acids, it is a typical complementary protein. These underutilised legumes could offer substantial health-promoting properties and can be effectively utilised in functional food development. Rice beans were also studied for numerous bioactive functions, especially their phenolics and flavonoids, which were examined and reported to exert anti-inflammatory, anti-diabetic, antioxidant, metabolic health-regulating, and anti-cancer properties (Karami & Duangmal, 2023). Recently, in India, this crop has gained focus primarily because of its climate adaptability, high protein content with a good amino acid score, and as a support for the traditional farming system to achieve protein security, particularly in the Himalayas and the northeastern region including Assam, Manipur, Mizoram, and Nagaland (Bepary et al., 2017; Kaur et al., 2013).

Pulses contain several anti-nutritional factors (ANFs) that can retard protein digestibility and mineral bioavailability, such as phytic acid, tannins, oxalates, saponins, and enzyme inhibitors (Kumar et al., 2022). Studies have shown that different processing techniques, i.e., soaking, cooking, germination, fermentation, and autoclaving, can significantly enhance protein digestibility, reduce ANFs, and improve the amino acid profile of pulses (Gu et al., 2023). Nevertheless, rice beans are considered safe for direct intake because of their low content of hemagglutinins and protease inhibitors (Katoch, 2020). To reduce ANFs, various traditional and advanced processing methods (such as soaking, germination, pressure cooking, roasting, and freeze-drying) can be utilised. Germination of legumes stimulates the production of endogenous enzymes that degrade phytates and increase the number of BACs (López-Martínez et al., 2017; Sarkhel & Roy, 2022). Furthermore, these methods can also improve the sensory quality and nutrient bioavailability, along with the formation of active components in rice beans.

Metabolomics of legumes is a high-throughput approach that provides in-depth profiling of low-MW metabolites present or formed during different processing techniques, i.e., hydrothermal, fermentation, or drying processes (Llorach et al., 2019). These tools also allow the identification of BACs, including flavonoids, polyphenols, organic acids, and amino acids, along with the evaluation of alterations that occur during processing (Lal et al., 2026). Mass spectrometry, particularly LC-MS, is a powerful foodomics tool for profiling metabolite changes during grain processing. It enables the comprehensive profiling of processing-induced metabolite changes, facilitating the identification of BACs, processing-related biomarkers, and the biochemical pathways associated with these transformations.

Despite extensive research on individual processing effects in major legumes, no study has comprehensively compared the metabolomic, structural, and functional consequences of diverse hydrothermal and thermal treatments in rice beans using untargeted HR-MS metabolomics integrated with physicochemical and spectroscopic analyses. The present study addresses this gap by (i) establishing distinct metabolic fingerprints for five processing treatments; (ii) identifying optimal processing strategies for specific food applications*.*

## 2 Materials and methods

### 2.1 Materials and reagents

Analytical-grade chemicals, including sodium hydroxide, sodium nitrite, aluminum chloride, folin-ciocalteu reagent (FCR), sodium carbonate anhydrous, gallic acid, catechin, ethanol, and 2,2-diphenyl-1-picrylhydrazyl (DPPH), were obtained from HiMedia (Mumbai, India). LC-MS grade methanol, acetonitrile, phosphate buffer, and formic acid were procured from Sigma-Aldrich (Bengaluru, India). Ultrapure water was produced using a Milli-Q system (Merck Millipore, Chennai, India). Other consumables like 25 mm, 0.22 μm PVDF syringe filters, 2 mL amber chromatography vials, glass beakers, and 50 mL polypropylene Falcon tubes were purchased from HiMedia and Fisher Scientific. Approximately 5 kg of whole dried rice beans (variety: JCR-08-7) were collected from local farms in Mizoram, Meghalaya, and Assam between October 2024 and April 2025. The beans were manually sorted to remove dust, stones, and other foreign material before processing.

### 2.2 Processing of Rice bean

Rice beans (200 g per treatment) were subjected to five processing methods. For soaking (SK), beans were soaked in distilled water (1:5, *w*/*v*) at approximately 25 °C for 24 h, oven-dried at 60 °C for 2 h, milled, sieved (80 μm), and stored at −18 °C (Abd El-Hady & Habiba, 2003). Germination (GR) was performed by germinating the soaked beans (1:3, *w*/*v*) in the dark at approximately 90% relative humidity for 36 h, followed by drying at 60 °C, milling, sieving, and storage at −18 °C (Mamilla & Mishra, 2017). For pressure cooking (PC), soaked beans (1:2, *w*/*v*) were cooked at 121 °C (30 psi) for 15 min, dried, milled, sieved, and stored at −20 °C. Sand roasting (SR) involved roasting soaked, air-dried beans in preheated sand at approximately 180 °C for 2 to 5 min with continuous stirring, followed by separation, milling, sieving, and storage (Qureshi et al., 2023; Singh et al., 2026). Freeze-drying (FD) was carried out by freeze-drying soaked beans (1:3, *w*/*v*) at −55 °C and 0.1 mbar for 48 h (BUCHI FreeZone), followed by milling, sieving, and packaging in airtight bags (Farooq & Wani, 2025). Since soaking is the initial step in most legume processing operations, all treatments were initiated from pre-soaked beans, with SK serving as the hydrated control.

### 2.3 Analysis of physical properties

#### 2.3.1 Seed dimensions and geometric properties

The length (L), width (W), and thickness (T) of randomly selected seeds were measured using a vernier calliper. The arithmetic mean diameter (AMD), geometric mean diameter (GMD), square mean diameter (SMD), and equivalent diameter (EQD) were calculated according to standard equations (Mohsenin, 1986).

#### 2.3.2 Sphericity (ϕ)

The sphericity of the treated rice beans was calculated using Eq. 1:

(1) $$\phi =\frac{\mathrm{GMD}}{L}$$

#### 2.3.3 Surface Area (S)

The surface area of the treated beans was determined from L, W, and T measurements of 20 randomly selected seeds per sample (Qureshi et al., 2023).

#### 2.3.4 Thousand kernel mass (M1000)

One hundred seeds were weighed using an electronic balance (±0.001 g), and the weight was multiplied by 10 to obtain the 1000-seed mass (Farooq & Wani, 2025).

#### 2.3.5 Bulk Density (ρb)

The bulk density of treated rice beans was determined using a 250 mL measuring cylinder:

(2) $$\rho_{b}=\frac{M}{V_{b}}$$

where Mis the sample mass (kg) and $V_{b}$is the bulk volume (m^3^) (Sharanagat & Goswami, 2014).

#### 2.3.6 True Density (ρt)

Ten grams of seeds were submerged in toluene in a graduated cylinder (±0.1 mL). The displaced volume was used to calculate the true density.

#### 2.3.7 Porosity (ε)

The porosity of treated rice beans was calculated using Eq. 3:

(3) $$\varepsilon =\frac{\rho_{t}-\rho_{b}}{\rho_{t}}\times 100$$

#### 2.3.8 Compressibility index (CI) and Hausner ratio (HR)

The Carr's index or compressibility index evaluates the flowability of the bean powder and is calculated using bulk and tapped densities (Sen et al., 2026)

(4) $$\mathrm{CI}=100\times \frac{\rho_{t}-\rho_{b}}{\rho_{t}},\mathrm{HR}=\frac{\rho_{t}}{\rho_{b}}$$

#### 2.3.9 Colour analysis

The surface colour of the different treated bean samples was evaluated using a Hunter Lab colorimeter (Hunter Lab Flex Ez, Hunter Associates Laboratory Inc., Reston, Virginia, U.S.A.) after being standardized using Hunter Lab colour standards and ‘L*’ (lightness), ‘a*’ (redness to greenness), and ‘b*’ (yellowness to blueness) values (Farooq & Wani, 2025). The browning index (BI) was calculated for each treatment.

### 2.4 Compositional analysis

The proximate composition of rice bean flour was determined using standard AOAC methods. Moisture content was measured by oven drying at 105 ± 5 °C to constant weight (AOAC, 2019). Crude protein was determined by the Kjeldahl method (AOAC, 2005), and nitrogen content was converted to protein using a conversion factor of 6.25. Total carbohydrate content was estimated using the Anthrone method (AOAC, 2005). Crude fat content was determined using the Soxhlet extraction method with a SoxCap and Soxtec system (Foss UK Ltd., UK) according to AOAC (2019), while ash content was determined by dry ashing at 550 °C for 3 h in a muffle furnace (AOAC, 2005). Moisture, protein, and ash contents were calculated using standard AOAC equations.

### 2.5 Functional properties

#### 2.5.1 Swelling index

The swelling index of the treated bean powder was determined using the method described by Neji et al. (2022). Samples (0.2 g) were placed in pre-weighed centrifuge tubes, and 10 mL of distilled water was added to each tube. The tubes were incubated in a water bath at temperatures of 50 °C, 60 °C, 70 °C, 80 °C, and 90 °C for 30 min with intermittent vortexing every 5 min. The tubes were then cooled to room temperature and centrifuged at 3000 rpm for 10 min. The swelling index was calculated as the ratio of the weight of the swollen sediment to the initial dry weight of the sample.

#### 2.5.2 Water absorption index (WAI) and water solubility index (WSI)

Water absorption index (WAI) and water solubility index (WSI) were determined according to Bashir and Aggarwal (2016). A 2 g sample was dispersed in 100 mL of distilled water (∼25 °C) for 30 min and heated at 90 °C for 15 min in a water bath. The cooked paste was centrifuged at 3000 ×*g* for 10 min. The supernatant was collected, and the dry solid content was determined by overnight evaporation at 110 °C. The WAI and WSI were calculated using Eqs. (5) and (6):

(5) $$\mathrm{WAI}\;\left(g/g\right)=\frac{\text{Weight of sediment}}{\text{Weight of sample}}$$

(6) $$\mathrm{WSI}\;\left(g/g\right)=\frac{\text{Weight of dissolved solids in supernatant}}{\text{Weight of sample}}$$

#### 2.5.3 Water absorption capacity (WAC)

The water absorption capacity (WAC) was determined according to Bashir and Aggarwal (2016). A 3 g sample was weighed and dispersed in 25 mL of distilled water (25 °C, 30 min) in a pre-weighed centrifuge tube. The samples were then centrifuged at 3000 rpm for 25 min. The supernatant was decanted, and the tube with the sediment was dried in a hot air oven for 25 min at 50 °C. The WAC was calculated using Eq. (7):

(7) $$\mathrm{WAC}\;\left(g/g\right)=\frac{\text{Final weight of sediment}-\text{Initial weight of sample}}{\text{Initial weight of sample}}$$

#### 2.5.4 Foaming capacity (FC) and foaming stability (FS)

Foaming capacity and stability were determined according to Farooq and Wani (2025). A 2 g sample was dispersed in 100 mL distilled water in a capped tube. The sample was shaken and agitated for 5 min using a hand blender (SS Hand Blender, Kent RO System Ltd., India), and the content was immediately poured into a 250 mL graduated cylinder. The volume of foam produced was observed at 0, 10, and 30 min. FC and FS were calculated using Eqs. (8) and (9):

(8) $$\mathrm{FC}\;\left(\%\right)=\frac{\text{Foam volume}\;\mathrm{at}\;0\;\min}{\text{Initial volume}}\times 100$$

(9) $$\mathrm{FS}\;\left(\%\right)=\frac{\text{Foam volume}\;\mathrm{at}\;\text{time}\;t}{\text{Initial foam volume}}\times 100$$

#### 2.5.5 Oil absorption capacity (OAC)

The oil absorption capacity was determined according to Qamar et al. (2020) with minor modifications. Each treated sample (2.5 g) was placed in a pre-weighed centrifuge tube and mixed with 20 mL of edible oil. The mixture was vortexed for 1 min and allowed to stand for 30 min at 25 °C, with intermittent stirring every 10 min. The tubes were then centrifuged at 3000 rpm for 10 min. The supernatant oil was decanted, and the tube was inverted for 10 min to drain excess oil. The gain in weight was expressed as OAC (g oil/g sample).

#### 2.5.6 Emulsion capacity (EC) and emulsion stability (ES)

Emulsion capacity and stability were determined according to Qamar et al. (2020). Each sample (0.2 g, dry basis) was placed in graduated centrifuge tubes, and 20 mL of distilled water and 20 mL of oil were added. The mixture was homogenised to obtain the emulsion, and the tubes were centrifuged at 2000 rpm for 5 min. The ratio of the height of the emulsion layer to the total height of the mixture was expressed as the emulsion capacity, calculated using Eq. (10):

(10) $$\mathrm{EC}\;\left(\%\right)=\frac{\text{Height of emulsion layer}}{\text{Total height of mixture}}\times 100$$

Emulsion stability was evaluated after heating the tubes at 80 °C for 30 min, calculated using Eq. (11):

(11) $$\mathrm{ES}\;\left(\%\right)=\frac{\text{Height of emulsion layer after heating}}{\text{Height of emulsion layer before heating}}\times 100$$

### 2.6 Analysis of antioxidant activity

#### 2.6.1 Extraction process of samples

Bioactive compounds were extracted from the processed rice bean samples using methanol-assisted extraction with minor modifications to the protocol described by Ferreira et al. (2022). Briefly, 1.0 g of each treated rice bean flour sample was accurately weighed into a 50 mL centrifuge tube, followed by the addition of 10 mL of methanol (HPLC grade). The mixture was homogenised using a vortex mixer (Tarsons Spinix Vortex Shaker, India) operating at 50 Hz for 2 min to ensure thorough dispersion. The samples were subsequently incubated at 4 °C for 12 h to facilitate optimal extraction of phenolic and flavonoid compounds. Following the incubation period, the mixtures were centrifuged at 3000 rpm for 30 min at room temperature. The supernatant was carefully collected, transferred to sterile centrifuge tubes, sealed with paraffin film to prevent evaporation and contamination, and stored at −18 °C until further analysis.

#### 2.6.2 Total phenolic content (TPC)

Total phenolic content was determined using the Folin-Ciocalteu colourimetric method with slight modifications (Aggarwal & Verma, 2025; Farooq & Wani, 2025). An aliquot of 200 μL of the methanolic extract was transferred to a 15 mL test tube, to which 1.5 mL of FCR (10-fold dilution with distilled water) was added. The contents were vortexed thoroughly and allowed to equilibrate for 5 min at room temperature. Subsequently, 1.5 mL of sodium carbonate solution (60 g/L) was added to neutralise the reaction mixture, followed by incubation at room temperature for 90 min in the dark to allow complete colour development. The absorbance of the resulting blue-colored complex was measured spectrophotometrically at 725 nm against a methanol blank. A standard calibration curve was prepared using gallic acid (concentration range: 20–100 μg/mL), and total phenolic content was expressed as milligrams of gallic acid equivalents per gram of sample (mg GAE/g). The TPC was calculated using Eq. (12):

(12) $$\mathrm{TPC}\;\left(\mathrm{mg}\;\mathrm{GAE}/g\right)=\frac{\left(A_{\mathrm{sample}}-b\right)}{a}$$

where *A*_*sample*_ represents the absorbance of the sample extract, a denotes the slope of the standard calibration curve (*y = ax + b*), and b is the y-intercept of the standard curve.

#### 2.6.3 Antioxidant activity (AOA)

The antioxidant activity of the extracts was evaluated using the 2,2-diphenyl-1-picrylhydrazyl (DPPH) radical-scavenging assay, according to the method described by Lal et al. (2026), with minor modifications. A 100 μL aliquot of the filtered extract was reacted with 3.9 mL of freshly prepared DPPH solution (6 × 10^−5^ mol/L in methanol) in a 5 mL volumetric flask. The reaction mixture was vortexed thoroughly and incubated in the dark at room temperature for 30 min. The absorbance was measured spectrophotometrically at 515 nm against methanol as a blank. The initial absorbance (at 0 min) and final absorbance (at 30 min) were recorded. The percentage of DPPH radical scavenging activity was calculated using Eq. (13):

(13) $$\%\text{DPPH radical scavenging activity}=\left(\frac{A_{0}-A_{S}}{A_{0}}\right)\times 100$$

where *A*₀ represents the absorbance of the blank (DPPH solution without extract), and *A*ₛ denotes the absorbance of the sample extract at 30 min.

#### 2.6.4 Total flavonoid content (TFC)

Total flavonoid content was determined using the aluminum chloride colourimetric method, as described by Lal et al. (2026), with minor modifications. An aliquot of 250 μL of the methanolic extract was transferred to a 10 mL volumetric flask and diluted with 1.25 mL of distilled water. Subsequently, 75 μL of sodium nitrite solution (5%, *w*/*v*) was added, and the mixture was allowed to stand for 5 min at room temperature to facilitate nitration. Subsequently, 150 μL of aluminum chloride solution (10%, *w*/*v*) was added, and the mixture was incubated for an additional 5 min to allow the formation of the flavonoid‑aluminum complex. Finally, 0.5 mL of sodium hydroxide solution (1.0 M) was added to develop the pink colour, and the total volume was made up to 5 mL with distilled water. The absorbance was measured spectrophotometrically at 510 nm against acidified methanol as a blank. Quercetin was used as the reference standard; a stock solution of 1 mg/mL was prepared in methanol, and working standards were prepared at concentrations ranging from 20 to 100 μg/mL. Total flavonoid content was expressed as milligrams of quercetin equivalents per gram of sample (mg QE/g). The TFC was calculated using Eq. (14):

(14) $$\mathrm{TFC}\;\left(\mathrm{mg}\;\mathrm{QE}/g\right)=\frac{\left(A_{\mathrm{sample}}-b\right)}{a}$$

where *A*_*sample*_ represents the absorbance of the sample extract, a denotes the slope of the standard calibration curve (*y = ax + b*), and b is the y-intercept of the standard curve prepared using quercetin standards.

#### 2.6.5 Fourier transform infrared spectroscopy (FT-IR)

FT-IR spectra of the processed rice bean samples were recorded using a PerkinElmer Spectrum spectrophotometer operating in transmission mode with KBr pellets. Spectral data were collected over the range of 400–4000 cm^−1^ at a resolution of 4 cm^−1^, with 2θ scans averaged at room temperature (Aggarwal et al., 2025). The obtained spectra were exported and processed using OriginPro 2024b (OriginLab Corporation, Northampton, MA, USA).

#### 2.6.6 X-ray diffraction (XRD)

X-ray diffraction (XRD) analysis was performed using a Malvern Panalytical X-ray diffractometer equipped with Cu Kα radiation (λ = 0.154 nm). The instrument was calibrated using silicon powder and operated at 45 kV and 40 mA. Data were collected over a 2θ range of 10°-85° at a scanning rate of 2°/min with a step size of 0.013° (Aggarwal et al., 2025).

#### 2.6.7 HR-MS-based bioactive profiling

Metabolomic profiling was performed using an untargeted high-resolution mass spectrometry (HR-MS) approach on a Thermo Orbitrap Eclipse Tribrid MS coupled with a Dionex UltiMate 3000 RSLC system. Briefly, 100 mg of each sample was extracted with 2.0 mL methanol: water (4:1, *v*/v), homogenised at 750 rpm for 30 min at 25 °C, centrifuged at 3500 rpm for 10 min, and filtered through a 0.22 μm PVDF syringe filter (Kumar et al., 2024). A 4 μL aliquot was injected onto a Hypersil GOLD™ C18 column (1.9 μm, 2.1 × 100 mm) at 40 °C with a flow rate of 300 μL/min using a gradient of water and methanol (both with 0.1% formic acid). Full-scan MS data were acquired in positive and negative ionisation modes over the *m*/*z* range of 100–1000 at a resolving power of 60,000, followed by HCD-MS/MS at normalised collision energies of 30%, 45%, and 60%. Pooled quality control (QC) samples were injected every 10 runs to monitor analytical reproducibility, and compounds with a coefficient of variation (CV) < 30% across QC injections were retained. Data processing was performed using Compound Discoverer 3.3.2.31 with S/N ≥ 3, deisotoping, and adduct deconvolution. Among the 10,533 raw spectral features detected, 283 metabolites were putatively annotated, representing the shared core metabolome across all treatments. These annotations remain tentative and are supported by accurate mass measurements (<5 ppm mass error) and MS/MS fragmentation patterns.

### 2.7 Statistical analysis

All experiments were conducted using three independently processed batches (biological replicates; *N* = 3), with each batch analysed once. Data are presented as mean ± standard deviation. One-way analysis of variance (ANOVA) was performed using Microsoft Excel software to determine significant differences among treatment groups, followed by Tukey's Honestly Significant Difference (HSD) post-hoc test for multiple comparisons at a probability level of *p* < 0.05.

## 3 Results and discussion

### 3.1 Proximate composition

The proximate composition of rice beans was significantly influenced by the different processing treatments applied. The moisture content was highest in SK (7.96%) and GR (7.82%) samples, primarily due to water uptake during hydration and sprouting processes. In contrast, FD (2.04%) and SR samples exhibited substantially lower moisture content, which may enhance storage stability and shelf life by reducing the risk of microbial growth. The ash content was highest in FD (4.2%) and SR (4.1%) samples, primarily due to the concentration of mineral constituents following extensive moisture removal. Meanwhile, PC (3.2%) and SK samples showed comparatively lower ash values, which may be attributed to the leaching of soluble minerals into the processing water, a phenomenon commonly reported in thermally processed legumes (Wang et al., 2010). Consistent with findings reported for processed chickpeas (Özcan et al., 2024), our results demonstrate that thermal and hydrothermal treatments significantly affect the mineral retention and functional properties of legume grains. Protein content varied considerably among treatments, ranging from 17.3% in SR to 24.3% in GR samples. The significantly higher protein content observed in germinated rice beans may be associated with enzymatic mobilisation of nitrogenous reserves, synthesis of new enzymatic proteins, and reduction in dry matter during sprouting (Sammoud et al., 2025; Xu et al., 2019). In contrast, lower protein content in SR and PC samples may be attributed to thermal denaturation, degradation, and leaching of soluble nitrogenous compounds during processing. Carbohydrate content ranged from 62.1% in SK to 63.2% in GR (Table 1). The lower carbohydrate content in SK may be attributed to the leaching of soluble sugars during soaking, whereas the higher value in GR may reflect dry-matter redistribution during sprouting. In contrast, FD and SR showed intermediate values of 62.6% and 62.2%, respectively, probably due to moisture removal and the resulting concentration effect. The carbohydrate reduction during sprouting may also be associated with amylolytic hydrolysis of starch into simpler sugars, which are utilised as energy sources during metabolic activation (Xu et al., 2019). Fat content showed no significant differences among treatments, ranging from 1.1% to 1.6%, with FD and SK samples exhibiting slightly higher values. Overall, the results indicate that GR and FD are effective processing methods for preserving or enhancing the nutritional composition of rice beans, whereas intensive thermal treatments, i.e., SR and PC, may reduce protein and mineral content due to leaching losses and heat-induced degradation.

**Table 1:** The proximate composition of different treatments of rice bean samples.

Treatments	Moisture %	Protein %	CHO %	Fat%	Ash %
SK	7.96 ± 0.12ᵃ	22.2 ± 0.4ᵇ	62.1 ± 1.3ᶜ	1.6 ± 0.3ᵃ	3.5 ± 0.1ᵃ
GR	7.82 ± 0.15ᵃ	24.3 ± 0.2ᵃ	63.2 ± 1.2ᵃ	1.2 ± 0.2ᵈ	3.4 ± 0.9ᵃ
SR	6.38 ± 0.10ᵇ	17.3 ± 0.6ᶜ	62.2 ± 1.1ᵇᶜ	1.3 ± 0.3ᶜ	4.1 ± 0.5ᵇ
FD	2.04 ± 0.20ᶜ	22.9 ± 0.2ᵇ	62.6 ± 1.2ᵃᵇ	1.1 ± 0.3ᵉ	4.2 ± 0.2ᵇ
PC	6.00 ± 0.50ᵇ	18.2 ± 0.2ᶜ	62.4 ± 1.4ᵇᶜ	1.4 ± 0.4ᵇ	3.2 ± 0.2ᵃ

Note: Different superscript letters (a–e) within the same column indicate significant differences (*p* ≤ 0.05). Values are expressed as mean ± SD (*n* = 3). CHO: Total carbohydrates; db: dry basis.

### 3.2 Engineering parameters

Hydrated samples (SK and PC) exhibited the highest axial dimensions and kernel mass, which may be attributed to moisture absorption and subsequent seed swelling. Similar increases in kernel dimensions and thousand-kernel weight with increasing moisture content have been reported for red kidney beans and cowpea seeds (Table 2) (Altuntas & Demirtola, 2007; Ravikumar et al., 2018). In contrast, dry-heat roasting (SR) and FD produced the smallest dimensions and lowest mass values, supporting earlier observations that dehydration processes proportionally reduce grain size and weight (Sharanagat et al., 2018).

**Table 2:** Effect of hydrothermal treatments on the physical properties of rice bean.

Sample	L (mm)	W (mm)	T (mm)	AMD (mm)	GMD (mm)	SMD (mm)	EQD (mm)	Φ (%)	SA (mm^2^)	BD (g/cm^3^)	TD (g/cm^3^)	P (%)	M (1000) g	CI (%)	HR
SK	13.69 ± 0.22^a^	5.8 ± 0.5^a^	5.11 ± 0.33^a^	7.38 ± 0.13ᵃ	8.20 ± 0.16ᵃ	13.36 ± 0.22ᵃ	28.94 ± 0.23ᵃ	58.30 ± 0.41ᶜ	63.66 ± 1.03ᵃ	0.212 ± 0.01ᵃ	2.137 ± 0.11ᵇ	90.1	453.1	90.08 ± 0.55ᵃ	10.08 ± 0.71ᵃ
GR	11.92 ± 0.51^b^	3.99 ± 0.24^b^	4.94 ± 0.12^a^	6.21 ± 0.11ᵇ	6.95 ± 0.12ᶜ	11.23 ± 0.17ᶜ	24.39 ± 0.21ᵇ	55.67 ± 0.38ᵈ	50.21 ± 0.88ᶜ	0.123 ± 0.01ᵃᵇ	2.688 ± 0.13ᵃ	95.5	330.6	95.42 ± 0.33ᵇ	21.85 ± 1.55ᵇ
SR	10.23 ± 0.24^c^	3.93 ± 0.19^c^	4.33 ± 0.32^b^	5.55 ± 0.09ᶜ	6.16 ± 0.14ᵈ	10.07 ± 0.15ᵈ	21.78 ± 0.19ᶜ	59.16 ± 0.42ᵇ	44.00 ± 0.77ᵈ	0.091 ± 0.00ᵇ	2.190 ± 0.10ᵇ	95.9	199.3	95.84 ± 0.25ᵇ	24.07 ± 1.70ᵇ
FD	12.37 ± 0.46^d^	5.38 ± 0.32^a^	4.77 ± 0.28^a^	6.82 ± 0.10ᵇ	7.50 ± 0.13ᵇ	12.29 ± 0.18ᵇ	26.61 ± 0.22ᵃ	59.60 ± 0.39ᵇ	59.58 ± 0.94ᵇ	0.166 ± 0.01ᵃᵇ	2.190 ± 0.12ᵇ	92.4	176.9	92.42 ± 0.42ᶜ	13.19 ± 0.93ᵃ
PC	10.73 ± 0.27^d^	6.28 ± 0.55^d^	5.58 ± 0.25^c^	7.30 ± 0.12ᵃ	7.48 ± 0.11ᵇ	12.65 ± 0.19ᵇ	27.43 ± 0.24ᵃ	68.85 ± 0.44ᵃ	60.57 ± 1.01ᵃ	0.194 ± 0.02ᵃ	1.066 ± 0.05ᶜ	81.7	287.1	81.50 ± 0.98ᵈ	5.49 ± 0.39ᶜ

Note: Different superscript letters (a-e) within the same column indicate significant differences (*p* ≤ 0.05). Values are expressed as mean ± SD (*n* = 3); L: length; W: width; T: thickness; AMD: arithmetic mean diameter; GMD: geometric mean diameter; SMD: squared mean diameter; ED: equivalent diameter; Φ: sphericity; SA: surface area; BD: bulk density; TD: true density; CI: compressibility index; HR: Hausner ratio; P: porosity mass; M (1000): thousand mass kernel; in the rows and columns with different superscripts are significantly different at *p* ≤ 0.05. Values expressed are mean ± Standard deviation.

Among the treatments, the highest sphericity was observed in the PC samples, primarily due to uniform moisture-induced expansion, which promoted a more rounded kernel geometry. Comparable effects have been observed in hydrothermally processed grains, where water uptake enhances seed roundness and dimensionality (Dharmaraj et al., 2015). Meanwhile, the lowest sphericity observed in the GR samples may be associated with enzymatic activity during sprouting, which causes elongation and surface irregularities in the seed structure (Bepary et al., 2018). Similarly, the highest surface area values in SK and PC and the lowest in SR demonstrate the strong dependence of surface area on seed volume and moisture content, as previously reported for processed legumes and grains (Abubakar et al., 2023; Sharanagat et al., 2018).

Regarding gravimetric properties, SK and PC exhibited higher bulk densities owing to increased mass accumulation per unit volume following hydration. In contrast, SR exhibited the lowest bulk density, which may be attributed to the formation of internal voids during moisture removal and thermal expansion. Similar reductions in bulk density have been documented in roasted chickpeas and other thermally processed legumes (Sharanagat et al., 2018; Qureshi et al., 2023). The highest true density observed in GR samples suggests compactness and reorganisation of the solid matrix during GR, potentially resulting from enzymatic modification of storage components. On the other hand, the lowest true density in PC samples (1.066 g/cm^3^) may be explained by starch gelatinisation and cellular disruption, which expand the internal granular structure and reduce the density of the solid fraction (Kittipongpatana et al., 2025; Ma et al., 2011).

Porosity values were highest in the SR, GR, and FD treatments, indicating substantial void formation associated with moisture loss and structural loosening during drying or GR. In contrast, the lowest porosity in the PC samples could be attributed to starch gelatinisation and the filling of intergranular spaces during hydrothermal treatment, consistent with previous observations in processed legume seeds (Jogihalli et al., 2017). Furthermore, the poor flow characteristics observed across all treatments, which indicate by Carr Index values higher than 80% and Hausner Ratio values exceeding 1.25, confirm the cohesive nature of legume flours. SR demonstrated the poorest flowability, whereas PC showed comparatively improved flow behaviour. These findings align with earlier reports suggesting that smaller and irregular particles exhibit higher cohesion and compressibility than larger and more uniform particles (Nkurikiye et al., 2023; Qureshi et al., 2023).

### 3.3 Colour analysis

Colour analysis revealed that processing significantly (*p* < 0.05) influenced the visual characteristics of rice bean flour (Table 4). The L* value (lightness) was highest in FD samples (86.16), indicating higher colour retention due to the low-temperature drying process, followed by SK (78.95) and SR (77.62) samples. In contrast, PC rice beans exhibited the lowest L* value (62.74), reflecting pronounced darkening caused by heat and moisture-induced browning reactions. The a* value (redness/greenness) was highest in GR flour (2.88 ± 0.01), possibly owing to enzymatic modifications and pigment transformation during GR, whereas FD samples showed a negative a* value (−0.62), indicating higher retention of green pigments. Similarly, the b* value (yellowness) was highest in GR (18.12) and lowest in PC (9.79), suggesting enhanced pigment development during GR and pigment degradation during thermal processing (Amanipour et al., 2024).

The overall colour difference (ΔE), calculated relative to the SK sample, was highest in PC (16.34), followed by FD (7.84), GR (6.92), and SR (1.60), indicating that PC produced the most pronounced visual changes. Overall, FD was the most effective treatment for preserving the native colour of rice bean flour, whereas GR enhanced the red and yellow colour attributes, and PC caused the highest colour deterioration due to thermal processing.

### 3.4 Functional properties

The functional properties of rice bean flour were significantly influenced (*p* < 0.05) by the applied processing methods, with GR and PC producing the most pronounced improvements. Among all treatments, PC exhibited the highest swelling index (5.31 g/g at 90 °C) (Table 3), which can be attributed to extensive starch gelatinisation during hydrothermal processing. Gelatinisation disrupts the crystalline structure of starch granules, facilitating water penetration, granule expansion, and swelling (Jogihalli et al., 2017). Similarly, the WAI was significantly higher (*p* < 0.05) in GR (3.20 g/g) and PC (3.06 g/g) than in the other treatments (Table 4), reflecting enhanced hydration capacity resulting from enzymatic degradation of starch and proteins during GR and the increased availability of hydrophilic groups following gelatinisation. The WSI followed a similar pattern, with GR (15.8 g/g) and PC (14.2 g/g), respectively, exhibiting the highest values owing to the formation of soluble carbohydrates, peptides, and other low-molecular-weight compounds during processing (Ghavidel & Prakash, 2006; Kemal et al., 2025).

**Table 3:** Swelling indices of hydrothermal treatments, sand roasting and freeze drying of rice bean samples.

Temperature (°C)	SK	GR	SR	FD	PC
50 °C	3.16 ± 0.05^a^	2.71 ± 0.06^b^	2.23 ± 0.07^c^	3.11 ± 0.14^a^	3.58 ± 0.08^d^
60 °C	3.40 ± 0.07^a^	3.22 ± 0.05^b^	2.66 ± 0.06^c^	3.47 ± 0.05^a^	3.94 ± 0.06^d^
70 °C	3.81 ± 0.08^a^	3.76 ± 0.07^b^	3.01 ± 0.08^c^	4.10 ± 0.06^a^	4.59 ± 0.07^d^
80 °C	4.52 ± 0.10^a^	4.35 ± 0.06^a^	3.19 ± 0.01^b^	4.43 ± 0.07^a^	5.06 ± 0.08^c^
90 °C	4.89 ± 0.09^a^	4.67 ± 0.08^a^	3.38 ± 0.05^b^	4.61 ± 0.09^a^	5.31 ± 0.09^c^

Note: Different superscript letters (a–d) within the same row indicate significant differences (*p* ≤ 0.05). Values are expressed as mean ± SD (*n* = 3).

**Table 4:** Different functional properties of rice bean samples treated with different methods.

Samples	L*	a*	b*	BI	WAI (g/g)	WSI (g/g)	WAC (g/g)	OAC (g/g)	EC (%)	ES (%)	FC (%)	FS (%)
SK	78.95 ± 0.33ᵇ	1.25 ± 0.42ᵇ	11.84 ± 0.11ᵇ	16.85	2.39 ± 0.03^a^	10.5 ± 0.2^a^	6.48 ± 0.01ᵃ	1.82 ± 0.03^a^	53.2 ± 0.13ᵇ	72.6 ± 0.01ᵇ	18.06 ± 0.32ᵇ	56.43 ± 0.02ᵇ
GR	76.53 ± 0.02ᵈ	2.88 ± 0.1ᵃ	18.12 ± 0.26ᵈ	28.92	3.20 ± 0.01^b^	15.8 ± 0.3^b^	8.63 ± 0.01ᵇ	2.28 ± 0.02^b^	58.9 ± 0.01ᵃ	76.3 ± 0.08ᵃ	32.82 ± 0.03ᵃ	70.02 ± 0.09ᵃ
SR	77.62 ± 0.05^c^	1.68 ± 0.23^ab^	12.61 ± 0.07^c^	18.46	2.77 ± 0.07^c^	9.1 ± 0.4^a^	7.44 ± 0.05ᶜ	2.42 ± 0.04^c^	49.5 ± 0.24ᶜ	66.1 ± 0.17ᶜ	8.23 ± 0.25ᶜ	28.23 ± 0.03ᶜ
FD	86.16 ± 0.11^a^	−0.62 ± 0.7^b^	9.38 ± 0.1^a^	11.65	2.26 ± 0.04^a^	12.6 ± 0.2^c^	6.21 ± 0.03ᵃ	2.06 ± 0.03^d^	55.4 ± 0.17ᵇ	74.1 ± 0.22ᵃᵇ	22.98 ± 0.06ᵇ	61.47 ± 0.10ᵇ
PC	62.74 ± 0.21^c^	1.53 ± 0.55^ab^	9.79 ± 0.5^a^	18.17	3.06 ± 0.01^b^	14.2 ± 0.3^b^	8.16 ± 0.09ᶜ	2.12 ± 0.03^c^	52.1 ± 0.02ᵇ	70.8 ± 0.05ᵇ	3.31 ± 0.14ᵈ	10.71 ± 0.07ᵈ

Note: L: lightness; a: redness–greenness; b: yellowness–blueness; BI: browning index; WAI: water absorption index; WSI: water solubility index; WAC: water absorption capacity; OAC: oil absorption capacity; EC: emulsion capacity; ES: emulsion stability; FC: foaming capacity; FS: foaming stability. Different superscript letters (a–e) within the same column indicate significant differences (*p* ≤ 0.05). Values are expressed as mean ± SD (*n* = 3).

The water absorption capacity (WAC) was highest in GR flour (8.63 g/g), primarily because GR activates hydrolytic enzymes that partially degrade storage macromolecules, exposing additional polar binding sites and increasing protein hydrophilicity (Amanipour et al., 2024). In contrast, the highest oil absorption capacity (OAC) was observed in SR flour (2.42 g/g), probably because dry-heat treatment exposed hydrophobic amino acid residues through partial protein denaturation, thereby enhancing lipid binding. Germinated flour also exhibited a relatively high OAC (2.28 g/g), suggesting that enzymatic modification of the protein matrix further improved its oil retention capacity (Jogihalli et al., 2017).

GR significantly improved the interfacial properties of rice bean flour. The highest foaming capacity (32.82%), foam stability (70.02%), emulsion capacity (58.9%), and emulsion stability (76.3%) were observed in GR flour (*p* < 0.05), reflecting increased protein solubility, improved molecular flexibility, and enhanced surface activity following partial proteolysis (Amanipour et al., 2024; Ghavidel & Prakash, 2006). In contrast, FD flour largely retained native protein functionality because of minimal thermal damage, whereas intensive thermal treatments, particularly PC and SR, reduced foaming and emulsifying properties owing to protein denaturation, aggregation, and reduced interfacial activity (Kemal et al., 2025). These findings are consistent with the FT-IR results, which indicated alterations in protein secondary structure through changes in the amide I and II regions (Fig. 4), and with the metabolomic profile, which revealed increased content of flavonoids, including quercetin, kaempferol, and naringenin in GR flour. Overall, GR was the most effective processing method for improving the techno-functional properties of rice bean flour, while PC mainly enhanced swelling and hydration, thereby increasing its suitability for value-added food applications.

### 3.5 Antioxidant activity

The bioactive and antioxidant properties of rice bean flour were significantly (*p* < 0.05) influenced by the applied processing methods, with GR and FD showing higher enhancement and retention of phytochemical compounds. Among all treatments, GR exhibited the highest total phenolic content (TPC) (8.42 mg GAE/g), total flavonoid content (TFC) (4.22 mg QE/g), and DPPH radical-scavenging activity (72.67%) (Table 5). These improvements were possibly attributed to the activation of the phenylpropanoid pathway during GR, which promotes the biosynthesis and release of phenolic compounds through enzymatic hydrolysis of bound phytochemicals (Cevallos-Casals & Cisneros-Zevallos, 2010; Dabeek & Marra, 2019). Similar increases in antioxidant activity have been reported in germinated legumes owing to enhanced accumulation of flavonoids and other secondary metabolites. FD bean samples also retained relatively high TPC (7.99 mg GAE/g) and TFC (3.99 mg QE/g), reflecting the ability of low-temperature sublimation to minimise thermal degradation and oxidative losses of thermolabile phytochemicals (Kessler et al., 2022). In contrast, SK samples exhibited moderate TPC (3.06 mg GAE/g) and DPPH radical-scavenging activity (33.19%), indicating that SK preserved part of the native antioxidant capacity without substantially stimulating phenolic biosynthesis.

**Table 5:** Bioactive functional activity of rice bean samples.

Sample	TPC (mg GAE/g)	%DPPH	TFC (mg QE/g)
SK	3.06 ± 0.01ᶜ	33.19 ± 1.02ᶜ	1.52 ± 0.07^b^
GR	8.42 ± 0.11ᵃ	72.67 ± 2.09ᵃ	4.22 ± 0.12ᵃ
SR	2.58 ± 0.01ᵈ	27.40 ± 1.06ᵈ	1.28 ± 0.01^b^
FD	7.99 ± 0.56ᵇ	25.60 ± 1.01ᵉ	3.99 ± 0.11^a^
PC	2.32 ± 0.01ᵉ	22.90 ± 0.02ᶠ	1.16 ± 0.01^c^

Note: TPC, total phenolic content; TFC, total flavonoid content; DPPH, 2,2-diphenyl-1-picrylhydrazyl radical scavenging activity. Different superscript letters (a–e) within the same column indicate significant differences (*p* ≤ 0.05). Values are expressed as mean ± SD (*n* = 3).

Thermal treatments, including SR and PC, significantly (*p* < 0.05) reduced the bioactive potential of rice bean flour. SR and PC exhibited lower phenolic content (2.58 and 2.32 mg GAE/g, respectively), flavonoid content (1.28 and 1.16 mg QE/g, respectively), and DPPH radical-scavenging activity (27.40% and 22.90%, respectively). These reductions are primarily associated with thermal degradation, oxidation, and leaching of phenolic compounds during high-temperature processing, which decreases the availability of antioxidant-active constituents (Koushki et al., 2023; Sharma et al., 2022).

Interestingly, although FD samples exhibited higher TPC and TFC than SK samples, they showed lower DPPH radical-scavenging activity (25.60% vs. 33.19%). This discrepancy suggests that antioxidant activity depends not only on the total phenolic content but also on the composition and reactivity of individual phenolic compounds. Therefore, TPC and TFC values do not necessarily correlate directly with DPPH radical-scavenging activity. Overall, GR was the most effective processing method for enhancing the phytochemical and antioxidant properties of rice bean flour, while FD effectively preserved phenolic compounds. In contrast, intensive thermal treatments significantly reduced bioactive constituents and antioxidant activity, highlighting the importance of selecting appropriate processing methods to maximise the nutritional and functional quality of rice bean flour.

### 3.6 Metabolomic profile of treated Rice bean samples

Untargeted high-resolution mass spectrometry (HR-MS)-based metabolomic profiling detected 10,533 metabolite features across the five rice bean processing treatments (Fig. 1 and Table 6). Among the treatments, GR showed the most diverse metabolomic profile, with 2851 metabolite features, followed by PC (2300), FD (2167), SK (1655), and SR (1596). A total of 283 metabolites were common across all treatments, representing the core rice bean metabolome, while treatment-specific metabolites accounted for the observed differences. Of these core metabolites, 155 were putatively annotated and are presented in Table 6. Treatment-dependent metabolite patterns were explored using unsupervised PCA and hierarchical clustering. Principal component analysis (PCA) revealed distinct treatment-specific clustering patterns, with PC1 and PC2 explaining 20.47% and 20.35% of the total variance, respectively (Fig. 3B). Although the first two components accounted for 40.82% of the total variance, the clear separation of treatments indicates that each processing method generated a distinct metabolic fingerprint. The moderate variance explained reflects the complexity and high dimensionality of the untargeted metabolomic dataset, with additional variation distributed across higher-order principal components. Hierarchical clustering further confirmed treatment-specific metabolite patterns, demonstrating that each processing method generated a distinct metabolic fingerprint.

**Fig. 1:**
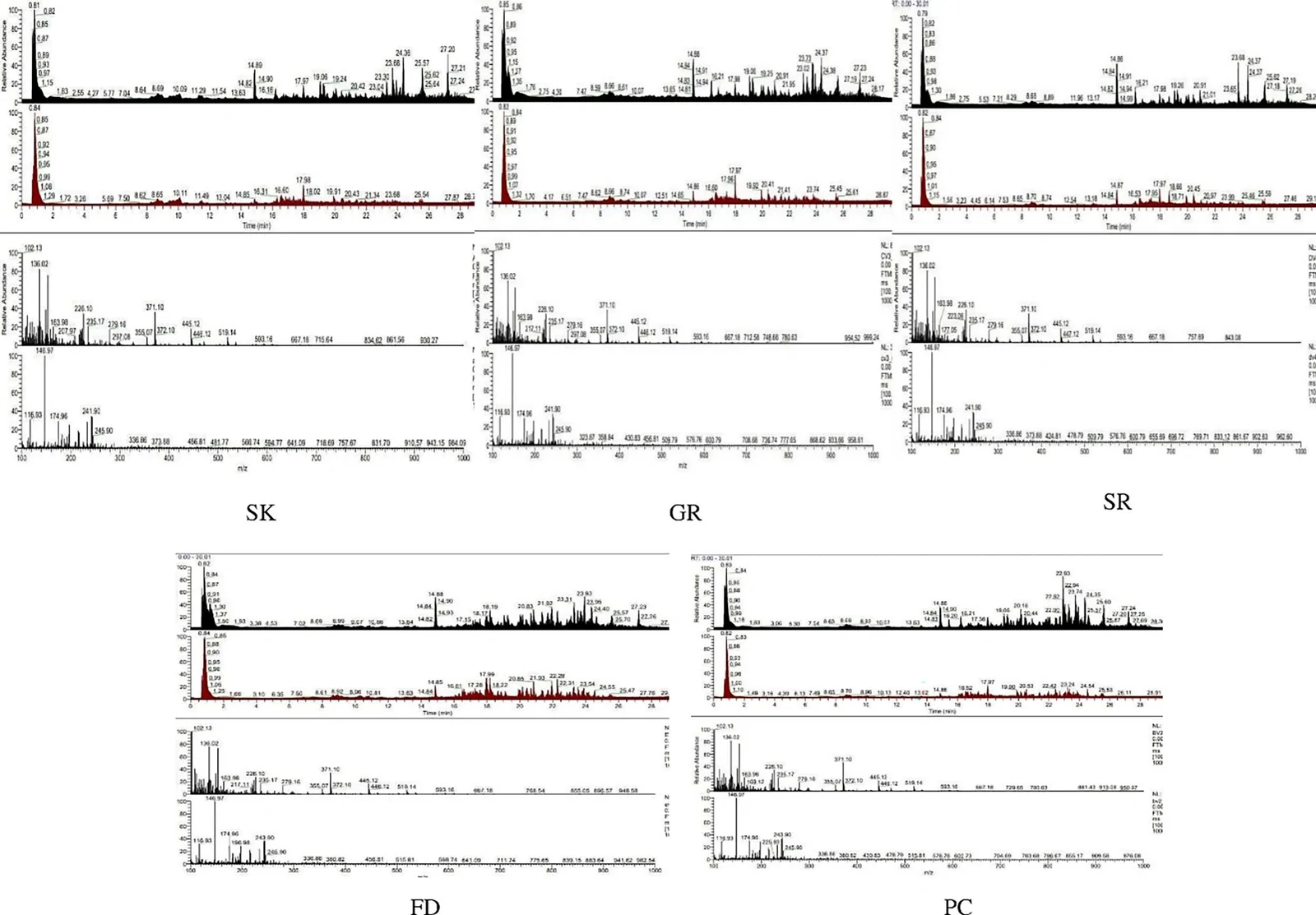
Representative base-peak chromatograms (BPC) from HR-Orbitrap-MS analysis of rice bean processed by soaking (SK), germination (GR), pressure cooking (PC), sand roasting (SR), and freeze-drying (FD). Chromatograms were acquired in positive ESI mode over a 30 min reversed-phase gradient (5–90% methanol with 0.1% formic acid).

**Table 6:** The major metabolites obtained from the LC-Orbitrap-MS metabolomics of rice bean samples.

SK: Soaking							
Metabolite Name	Formula	Annoted Delta Mass [ppm]	Calc.MW	*m*/*Z*	RT [min]	Area (Max.)	Metabolite Class
L-Histidine	C_6_H_9_ N_3_O_2_	0.16	155.06	156.07	0.738	25,073,918	AA
Acetylglucosamine	C₈H₁₅NO₆	3.30	221.08	606.07	0.722	26,575,656	Sugar
Glucose-1-phosphate	C_6_H_13_ O_9_P	1.65	260.03	259.02	0.738	11,444,968	Sugar
L-Arginine	C_6_H_14_N_4_O_2_	0.38	174.11	175.10	0.75	2,147,208	AA
α-Trehalose	C_12_H_22_O_11_	2.04	342.11	341.10	0.795	81,742,308	Sugar
Ferulic acid	C_10_ H_10_ O_4_	2.42	194.05	193.05	9.77	1,995,003	Phenolic
Asparagine	C_4_ H_8_ N_2_ O_3_	0.85	132.05	133.06	0.836	2,321,108	AA
Catechin	C_15_ H_14_ O_6_	0.58	290.07	291.08	1.02	1,864,209	Flavonoid
Proline	C_5_ H_9_ N O_2_	0.92	115.06	116.07	0.843	3,264,108	AA
Aminocyclohexanecarboxylic acid	C_4_ H_7_ N O_2_	0.68	101.04	143.08	0.843	2,082,308	Non-proteinogenic AA
Geniposide	C_17_ H_24_ O_10_	3.61	388.13	387.13	7.661	3,392,161	Glycoside
3′,5,7-Trihydroxy-4′-methoxyflavanone	C_16_ H_14_ O_6_	2.68	302.07	301.07	14.61	1,318,141	Flavonoid
Itaconic acid	C_5_ H_6_ O_4_	1.73	130.02	111.00	0.9	20,127,109	Organic acid
Nicotinamide	C_6_ H_6_ N_2_ O	0.88	122.04	123.05	0.919	72,578,947	Vitamin derivative
L-Isoleucine	C_6_ H_13_ N O_2_	0.88	131.09	132.10	0.971	9,082,108	AA
Gluconic acid	C_6_ H_12_ O_7_	1.9	196.05	195.05	1.027	15,336,602	Sugar acid
Dianthoside	C_12_ H_16_ O_8_	0.53	288.08	289.09	1.032	57,145,847	Glycoside
Dimethoxy furanoflavanone	C_19_ H_16_ O_5_	0.73	324.11	357.13	15.	5,879,353	Flavonoid
Bakuchalcone	C_20_ H_20_ O_5_	2.28	340.13	339.12	18.26	1,467,059	Flavonoid
**GR: Germination**							
Kaempferol	C_15_ H_10_ O_6_	0.08	286.04	287.05	12.69	12,016,218	Flavonoid
Salicylic acid	C_7_ H_6_ O_3_	0.46	138.03	139.03	1.22	60,450,625	Phenolic
Catechin	C_15_ H_14_ O_6_	2.58	290.07	289.07	1.41	61,722,375	Flavonoid
Quercetin	C_15_ H_10_ O_7_	0.4	302.04	303.05	11.82	47,404,358	Flavonoid
Naringenin	C_15_ H_12_ O_5_	1.94	272.06	271.06	13.39	16,433,121	Flavonoid
L-Isoleucine	C₆H₁₃NO₂	2.43	131.09	132.11	1.99	25,201,554	AA
N-Palmitoyl Glycine	C_18_H_35_NO_3_	1.19	313.26	312.25	23.99	9,438,671	FA
4-Methylphenol	C_7_H_8_O	1.64	108.05	107.05	1.35	8,642,282	Phenolic
2,6-di-tert-butylphenol	C_15_H_24_O	0.02	206.16	207.17	21.94	10,051,757	Phenolic
Ferulic acid	C₁₀H₁₀O₄	1.87	194.05	193.05	10.56	3,283,606	Phenolic
Nicotinamide	C_11_H_12_N_2_O_3_	0.43	122.04	123.05	1.055	38,197,516	Vitamin derivative
Di-tert-butylphenol	C_6_H_6_N_2_O	0.02	206.17	207.17	21.94	10,051,757	Phenolic
Butyl-4-methoxyphenol	C_14_H_22_O	2.23	180.11	179.10	15.79	1,245,872	Phenolic
3-Hydroxyaminophenol	C_10_H_14_O	1.99	125.04	124.04	1.31	34,106,723	Phenolic
Oleic acid	C_18_ H_34_ O_2_	2.07	282.25	281.24	25.52	44,231,205	FA
**SR: Sand Roasted**							
Ferulic acid	C_10_ H_10_ O_4_	2.87	194.05	433.11	9.94	87,267,099	Phenolic
Gentisic acid	C_7_ H_6_ O_4_	2.48	154.02	153.01	1.296	83,738,081	Phenolic
Catechin	C_15_ H_14_ O_6_	0.55	290.07	291.08	7.63	51,542,015	Flavonoid
Coumarin	C_9_ H_6_ O_2_	0.63	146.03	147.04	11.46	23,679,090	Coumarin
Kaempferol	C_15_ H_10_ O_6_	0.14	286.04	287.05	13.15	21,062,039	Flavonoid
Genistein	C_15_ H_10_ O_5_	0.85	270.05	271.06	15.01	92,063,144	Isoflavonoid
Daidzein	C_15_ H_10_ O_4_	0.25	254.05	255.06	13.77	63,367,834	Isoflavonoid
Isorhamnetin	C_16_ H_12_ O_7_	0.49	316.05	317.06	12.99	6,426,577	Flavonoid
Hispidulin	C_16_ H_12_ O_6_	2.68	300.06	299.05	15.19	94,183,188	Flavonoid
Glycitin	C₁₆H₁₂O₅	0.34	284.07	447.12	10.10	3,719,582	Isoflavonoid
Genistin	C₂₁H₂₀O₁₀	1.10	432.10	433.11	12.40	5,182,113	Isoflavonoid
Hyperoside	C_15_ H_14_ O_6_	0.86	464.09	465.10	11.64	8,973,625	Flavonoid
Tryptophan	C_11_ H_12_ N_2_ O_2_	2.89	204.09	203.08	1.789	42,598,261	AA
Arginine	C_6_ H_14_ N_4_ O_2_	0.04	174.11	175.11	0.74	351,703,285	AA
Lupeol	C_30_ H_50_ O	0.95	426.38	427.39	24.83	4,623,708	Triterpenoid
Maltol	C_6_ H_6_ O_3_	1.27	126.03	127.03	1.914	155,628,465	Flavor compound
Maritimetin	C_15_ H_10_ O_6_	1.17	286.04	285.04	13.49	85,602,217	Flavonoid
Methyl-2-aminobenzoate	C_9_ H_13_ N O_4_	0.18	199.08	200.09	1.062	31,695,653	Ester
Methyl 9-butylperoxy-10,12-octadecadienoate	C_23_ H_42_ O_4_	0.15	382.30	383.31	25.40	4,110,063	Ester
**FD: Freeze Dried**							
Coumarin	C_9_H_6_O_2_	−0.44	146.03	147.04	10.05	37,361,540	Coumarin
Daidzein	C_15_H_10_O_4_	0.07	254.05	255.06	13.77	85,157,412	Isoflavonoid
Tryptophan	C_11_H_12_N_2_O_2_	3.19	204.09	203.08	9.213	10,487,385	AA
Eicosapentaenoic acid	C_2_0H_30_O_2_	−0.77	302.22	303.23	19.47	4,479,658	FA
Ferulic acid	C_10_H_10_O_4_	−0.4	194.05	227.09	9.93	32,511,744	Phenolic
Fumaric acid	C_4_H_4_O_4_	2.79	116.01	115.01	9.065	13,256,927	Organic acid
Genistein	C_15_H_10_O_5_	0.48	270.05	271.05	15.001	11,861,150	Isoflavonoid
Genistin	C_21_H_20_O_10_	−0.21	432.10	433.11	11.153	6,736,758	Isoflavonoid
Gentisic acid	C_7_H_6_O_4_	2.48	154.02	153.01	1.264	6,880,253	Phenolic
Hispidulin	C_16_H_12_O_6_	2.88	300.06	299.05	15.929	2,407,393	Flavonoid
Hyperoside	C_21_H_20_O_12_	1.03	464.09	465.10	11.68	13,919,141	Glycoside
Isoliquiritigenin	C_15_H_12_O_4_	1.21	256.07	255.06	17.214	6,263,500	Flavonoid
Isorhamnetin	C16H12O7	1.34	316.05	317.06	15.89	5,314,698	Flavonoid
Itaconic acid	C5H6O4	1.89	130.02	111.00	0.841	268,143,053	Organic acid
Jasmone	C_11_H_16_O	−0.76	164.11	147.11	17.43	20,590,672	Oxylipid
Kaempferol	C_15_H_10_O_6_	0.09	448.10	449.10	12.77	5,001,308	Flavonoid
Quercetin	C_15_H_10_O_7_	0.19	302.04	303.04	11.70	58,245,073	Flavonoid
Linoleic acid	C_18_H_32_O_2_	−0.93	306.25	307.26	25.44	24,824,203	FA
**PC: Pressure Cooked**							
Hydroxyaminophenol	C_6_ H_7_ N O_2_	1.55	125.04	124.04	1.13	6,806,324	Phenolic
3′,5,7-Trihydroxy-4′-methoxyflavanone	C_16_ H_14_ O_6_	2.38	302.07	301.07	14.61	2,151,937	Flavonoid
2,6-Di-tert-butylphenol	C_14_ H_22_ O	0.39	206.16	207.17	21.46	5,068,744	Phenolic
Quinoline	C_9_ H_7_ N	0.58	129.05	130.06	19.49	40,087,515	Aromatic compound
Pinolenic acid	C_18_ H_30_ O_2_	0.17	278.22	279.23	17.97	10,381,289	FA
Tyromycic acid	C_30_ H_42_ O_3_	0.35	450.31	483.34	17.97	18,461,323	FA
Lupeol	C_30_ H_50_ O	−0.1	426.38	427.39	25.08	3,188,406	Terpenoid
Oleamide	C_18_ H_35_NO	−0.53	281.27	282.27	27.23	11,460,973	FA
Betulin	C_30_ H_50_ O_2_	0.46	442.38	425.37	24.88	21,533,458	Terpenoid
1-Linoleoyl glycerol	C_21_H_38_ O_4_	0.22	354.27	355.28	24.85	4,837,716	Lipid
Oleanolic acid	C_30_ H_48_ O_3_	−0.05	456.36	457.36	22.52	69,964,375	Terpenoid
3,5,6-Trimethoxy-3′,4′-methylene-dioxyfurano flavanone	C_21_ H_18_ O_8_	0.74	398.11	399.10	10.07	17,796,150	Flavonoid
Acetylglucosamine	C_17_H_27_N_3_O_17_P_2_	3.3	607.08	606.07	0.722	26,575,656	Sugar
Glucose 1-phosphate	C_6_ H_13_ O_9_ P	1.65	260.03	259.02	0.73	11,444,968	Sugar
L-Isoleucine	C_6_ H_13_ N O_2_	0.88	131.09	132.10	0.97	9,108,208	AA
Bakuchalcone	C_20_ H_20_ O_5_	2.28	340.13	339.12	18.26	1,467,059	Flavonoid

Note- Calc. MW: Calculated molecular weight; RT: Retention time; m/z: mass/charge; AA: Amino acid; FA: Fatty acid.

All molecular formulas and exact masses were verified against the PubChem and ChemSpider databases. Metabolite annotations were manually validated, and any discrepancies identified during data curation were resolved by re-interrogating the LC-MS/MS raw data against the mzCloud spectral library, thereby improving the confidence of compound identification.

A total of 283 metabolites were common to all treatments, representing the core rice bean metabolome, whereas treatment-specific metabolites accounted for the observed variation. SK samples retained catechin, itaconic acid, and L-histidine, whereas GR promoted the accumulation of flavonoids, including quercetin, kaempferol, and naringenin, consistent with enhanced phenylpropanoid metabolism during sprouting (Dabeek & Marra, 2019). In contrast, SR favoured the retention or formation of relatively thermostable phytochemicals (genistein, daidzein, and gentisic acid), while thermolabile compounds, including catechin, were reduced following thermal processing (Mechchate et al., 2021). FD samples retained heat-sensitive metabolites, including isorhamnetin, hyperoside, and coumarin, reflecting the mild processing conditions that minimise thermal degradation (Al Hasani et al., 2023). PC resulted in a distinct metabolite profile characterised by compounds like lupeol, quinoline, and pinolenic acid, primarily reflecting processing-induced chemical transformations (Elebiju et al., 2023; Ramasinghe & Xu, 2025).

The metabolomic changes were consistent with the observed functional properties of the processed flours. Germinated flour, which exhibited superior hydration, foaming, and emulsifying properties, also contained hydrophilic metabolites and flavonoids, including quercetin, kaempferol, and naringenin, consistent with the activation of phenylpropanoid metabolism. Although treatment-specific metabolite profiles were identified, pathway enrichment analysis was not performed and should be included in future studies to provide deeper mechanistic insights into processing-induced metabolic changes. Meanwhile, the higher oil absorption capacity of sand-roasted flour corresponded with FT-IR evidence of protein structural modification, suggesting increased exposure of hydrophobic binding sites following dry-heat treatment. Along with FT-IR and XRD results, these findings indicate that different processing methods induce distinct structural modifications that collectively influence the techno-functional properties of rice bean flour.

Overall, metabolomic analysis demonstrated that processing significantly altered the composition of rice beans. GR produced the highest enrichment of bioactive metabolites, whereas FD effectively preserved thermolabile compounds. In contrast, SR and PC generated distinct processing-dependent metabolite profiles. These findings provide molecular insights into the effects of processing and support the utilisation of processed rice bean flour as a nutritionally enriched ingredient for functional food applications.

### 3.7 Abundant metabolites

Metabolites were obtained from five treatment groups, followed by detailed analysis of the identified metabolites. A substantial quantity of metabolites was acquired, with the most abundant including sedanolide, p-cymene, pentamethylbenzene, 2, 4-dimethyl-1-vinylbenzene, and 9S, 13R-12-oxophytodienoic acid. Bicyclohexyliden-2-one, dendryphiellic acid, 4-formyl-2-hydroxy-4-hexamethyl-trihydroxy-6-hydroxymethyl-oxanyloxycarbonyl-icosahydropicenyl-oxy-trihydroxyoxane-2-carboxylic acid. Corchori fatty acid F, 9-Oxononanoic acid, Octyl methoxycinnamate, 13(*S*)-HOTrE, Bis (4-ethylbenzylidene) sorbitol, 10-hydroxy-heptamethyl-oxahexacyclo-tetracosenone, 19-norandrostenedione, 1-linoleoyl glycerol, 3-vinyltoluene, and ethylbenzene were the predominant compounds in FD. Various functional compounds, fatty acids, and secondary metabolites are typically identified in rice bean seeds. The results demonstrated that the identified seed metabolite groups mostly affect the variance of functional genes rather than the taxonomic composition. Seed metabolites facilitate survival under climatic circumstances. These categories of metabolites are the principal nutritional components provided by indoor-farmed crops (Fig. 2).

**Fig. 2:**
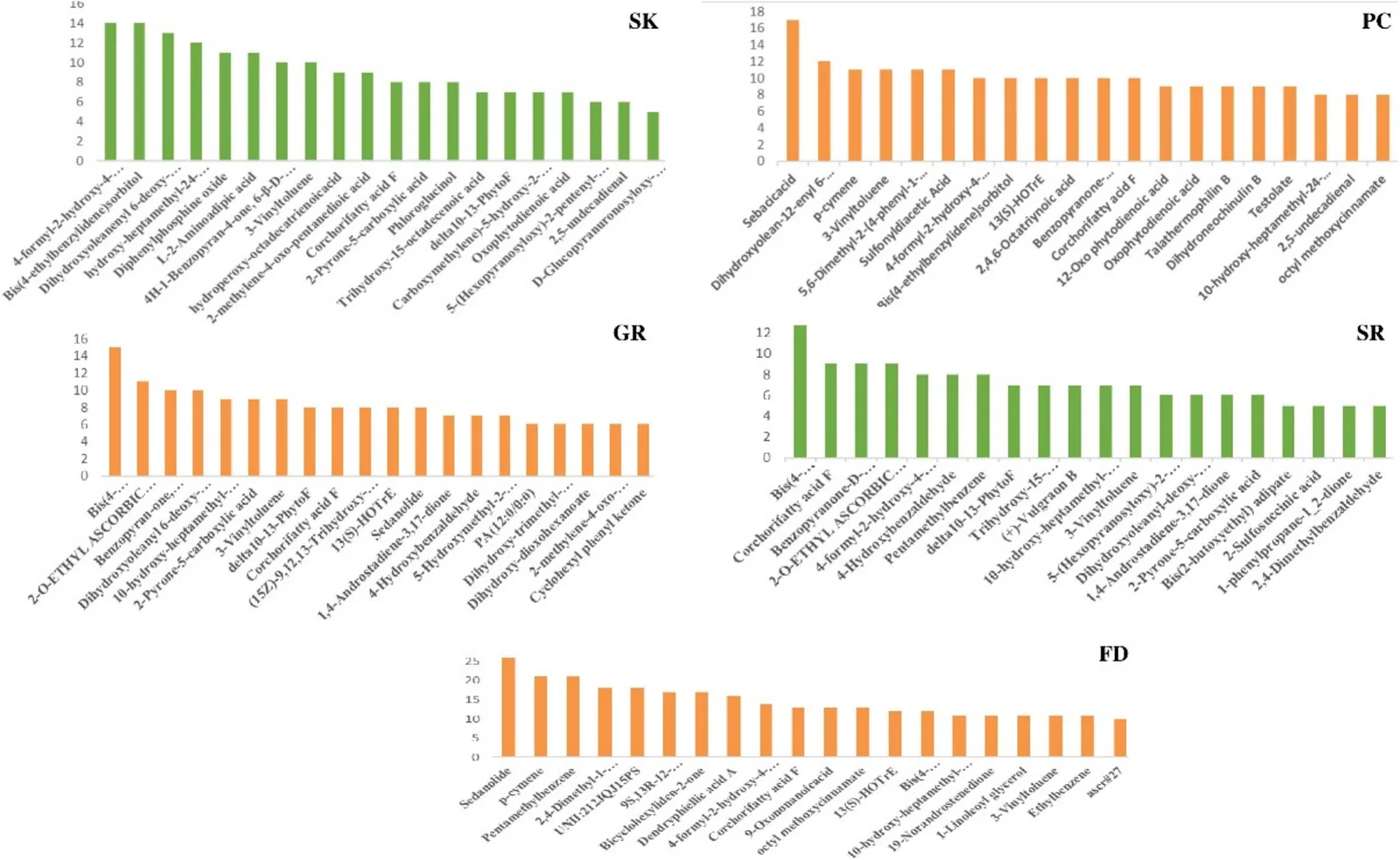
Bars represent key metabolites obtained in soaking (SK), pressure cooking (PC), germination (GR), sand roasting (SR), and freeze-drying (FD) of the rice bean sample.

In the PCA for metabolomics, an eigenvalue measures how much the original metabolites represented by each principal component differ from each other. PCA1 and PCA2 showed a clear difference between the five groups of rice bean seeds based on their eigenvalues. High eigenvalues indicate that the principal components that go with them make up a larger part of the overall variability. This makes it easier to choose the most important components for further analysis, which reduces the complexity of the data while keeping important metabolic patterns (Fig. 3A). The PCA score plot showed that the first three principal components accounted for PC1 (20.47%) and PC2 (20.35%). A heatmap created using Ward's hierarchical clustering method also showed that the clusters were separate. We used LC-MS/MS to examine the metabolite annotations of five rice bean seed treatments: SK, PC, GR, SR, and FD. PCA was performed to acquire a comprehensive overview of the raw metabolomics data and to verify instrument efficacy (Fig. 3C).

**Fig. 3:**
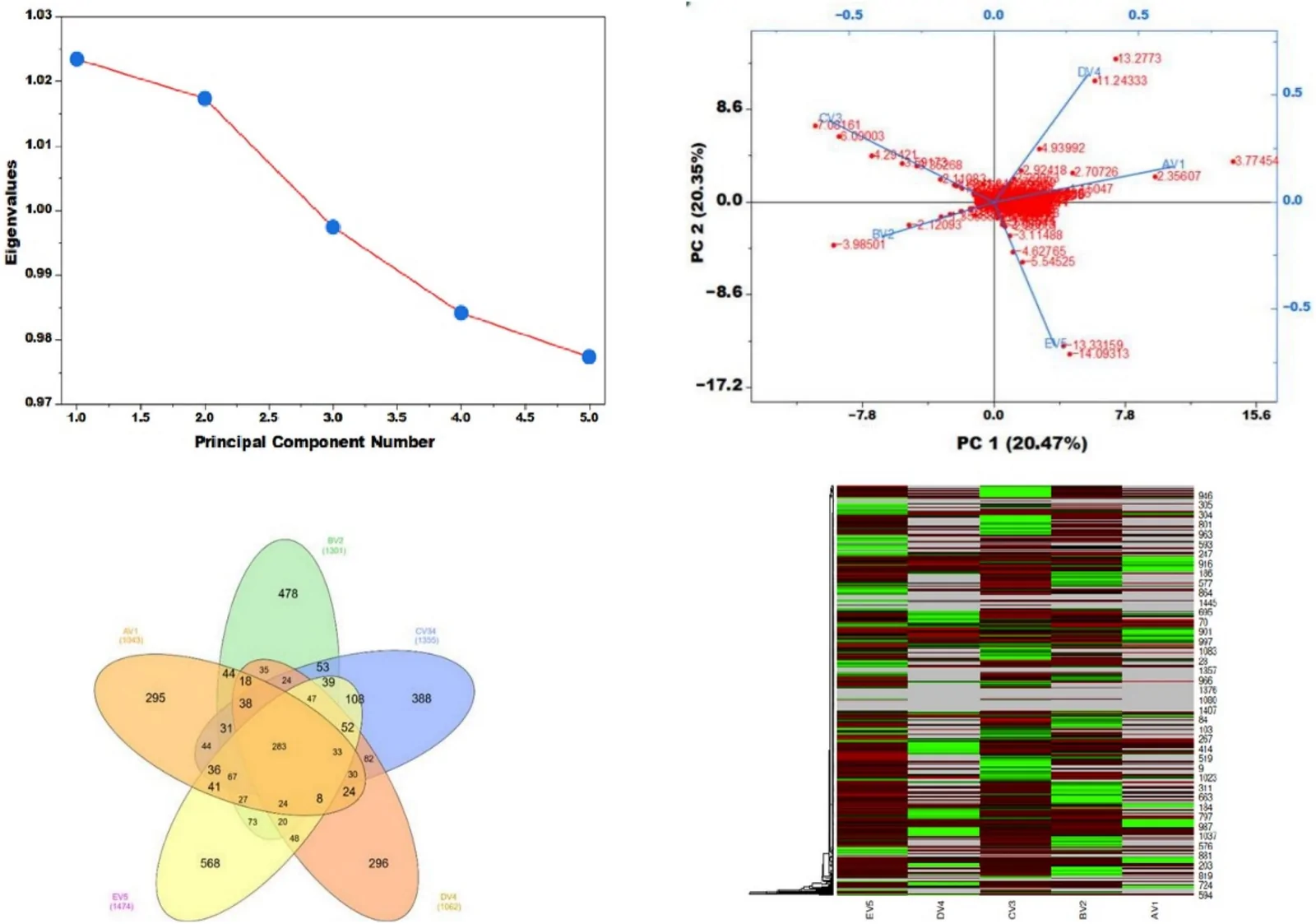
Multivariate metabolomic analysis of rice bean (*Vigna umbellata*) under five processing treatments: soaking (SK), germination (GR), pressure cooking (PC), sand roasting (SR), and freeze-drying (FD). **(A)** Screen plot of eigenvalues for the first five principal components derived from LC-Orbitrap-MS feature intensities. **(B)** PCA score and loading biplot; PC1 and PC2 accounted for 20.47% and 20.35% of the total variance, respectively. Blue score points represent individual biological replicates (independently processed batches); red arrows denote metabolite loading vectors. **(C)** Venn diagram illustrating the distribution of detected metabolites across the five treatments, showing unique and shared compound sets; a core metabolome of 283 compounds was common to all treatments. **(D)** Hierarchical clustering heatmap (Ward's method, Pearson correlation) of the top differentially abundant annotated metabolites. The colour scale indicates relative abundance: green = higher abundance; red/brown = lower abundance; black/grey = non-significant or baseline abundance. (For interpretation of the references to colour in this figure legend, the reader is referred to the web version of this article.)

The PCA plot illustrates a distinct disparity between the control and treatment groups, indicating divergent metabolic profiles. The separation was mostly attributed to metabolites, secondary metabolites, hormones, fatty acids, carbohydrates, and organic acids, consistent with the previous discussion regarding the comparison of these metabolites. Lipids are essential components of cell membranes, which serve as a source of energy for high-demand activities, and play a crucial role in numerous signal transduction pathways. This study analysed the relationships among the various metabolites found in the seeds of four different legume treatments.

Heatmap analysis was performed using Pearson's correlation on the datasets of four distinct groups: SK, PC, GR, SR, and FD. In the correlation matrix of groups SK, PC, GR, SR, and FD (Fig. 3D), thousands of metabolites were detected across all five groups. A majority of metabolites, including fatty acids, carbohydrates, and smaller compounds, predominantly secondary metabolites (anthocyanins and carotenoids), were found alongside numerous sugars, demonstrating advantageous relations.

Venn diagrams illustrated metabolite overlap among SK, PC, GR, SR, and FD, while hierarchical clustering heatmaps showed metabolite intensity patterns and relationships. The analysis highlighted metabolites associated with nitrogen metabolism, plant hormone biosynthesis, and defense responses. InteractiVenn was used to generate the Venn diagrams, identifying 283 metabolites common to all five groups.

### 3.8 FT-IR analysis

The FT-IR spectra provide comprehensive information regarding structural modifications in proteins, carbohydrates, and lipids induced by different processing treatments (Fig. 4). A broad absorption band observed around 3273 cm^−1^ in FD, PC, and SK samples corresponds to overlapping O—H stretching vibrations of hydroxyl groups from carbohydrates and absorbed water, along with N—H stretching of proteins. The presence of this broad band indicates intact intermolecular hydrogen bonding networks within these matrices. In contrast, SR and GR samples exhibited multiple sharp peaks within the 3746–3981 cm^−1^ region, which suggests disruption and rearrangement of hydrogen bonding interactions. Dry heat roasting promotes moisture removal and structural dehydration, whereas GR activates endogenous enzymes that modify protein-carbohydrate associations and expose previously bound functional groups (Dong et al., 2023; Dzurendová et al., 2021).

**Fig. 4:**
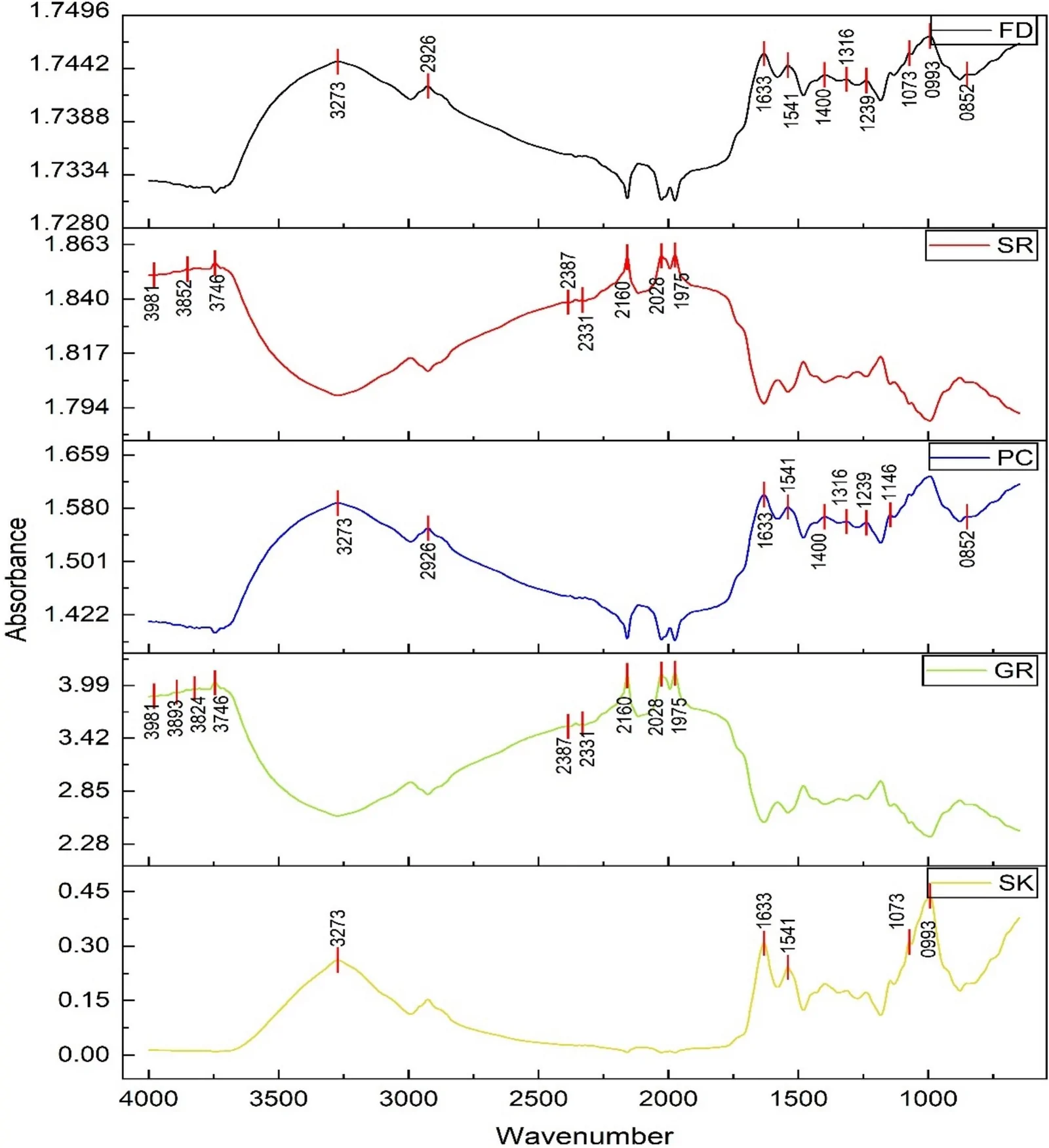
Fourier-transform infrared (FT-IR) spectra (400–4000 cm^−1^) of rice bean flour subjected to five processing treatments: soaking (SK), germination (GR), pressure cooking (PC), sand roasting (SR), and freeze-drying (FD).

In the aliphatic stretching region (2800–3000 cm^−1^), the prominent peak at approximately 2926 cm^−1^ corresponds to asymmetric CH₂ stretching vibrations associated with lipid chains and polysaccharide backbones. This peak remained well-defined in FD and PC samples, indicating the preservation of lipid and starch structural integrity under FD and moist heat conditions. However, the reduced intensity and slight shift observed in SR samples suggest thermal degradation or interaction between lipids and amylose chains, potentially resulting in amylose-lipid complex formation during high-temperature roasting (Dzurendová et al., 2021). Variations were observed in the protein fingerprint region (1500–1700 cm^−1^), which reflects conformational changes in protein structures. The amide I band centred near 1633 cm^−1^ arises primarily from C=O stretching vibrations and represents protein secondary structures (β-sheet conformations), while the amide II band around 1541 cm^−1^ corresponds to N—H bending and C—N stretching of peptide bonds. The increased intensity in PC samples indicates protein unfolding and denaturation caused by hydrothermal processing, which enhances exposure of peptide linkages. In GR samples, spectral modifications in this region are attributed to proteolytic degradation of storage proteins into smaller peptides and amino acids during sprouting. The distinct absorption bands observed at 2160 cm^−1^ and 1975 cm^−1^ in the sand-roasted (SR) samples may be associated with combination bands of carbonyl (C=O) stretching and C—H bending vibrations arising from heat-induced carbonyl-containing compounds. These spectral features are consistent with the formation of Maillard reaction intermediates and conjugated carbonyl structures during dry-heat processing (Tamanna & Mahmood, 2015). However, as FTIR alone cannot identify specific Maillard products, these assignments remain tentative and require confirmation through targeted chromatographic or mass spectrometric analyses. The carbohydrate fingerprint region (800–1200 cm^−1^) further demonstrates structural alterations in starch components. Characteristic peaks at 1073 cm^−1^ and 993 cm^−1^ correspond to C—O and C—C stretching vibrations of starch polysaccharides. Sharp spectral features retained in FD and SR samples indicate preservation of ordered crystalline starch structures. Meanwhile, broadening of these peaks in PC samples confirms starch gelatinisation resulting from the disruption of crystalline regions under combined heat and pressure treatment. GR rice bean samples exhibited noticeable spectral modifications due to activation of amylolytic enzymes, leading to hydrolysis of complex starch into simpler sugars and subsequent alteration of carbohydrate molecular organisation (Liu et al., 2024).

### 3.9 XRD analysis

XRD analysis indicated that rice bean starch predominantly exhibits an A-type crystalline polymorphic structure, characterised by prominent diffraction peaks at 2θ values of approximately 17°, 18°, and 23° (Fig. 5). These reflections are associated with the densely packed monoclinic unit cell arrangement commonly observed in cereal and legume starches, confirming the presence of a well-defined semi-crystalline organisation within the starch granules (Munjal et al., 2024).

**Fig. 5:**
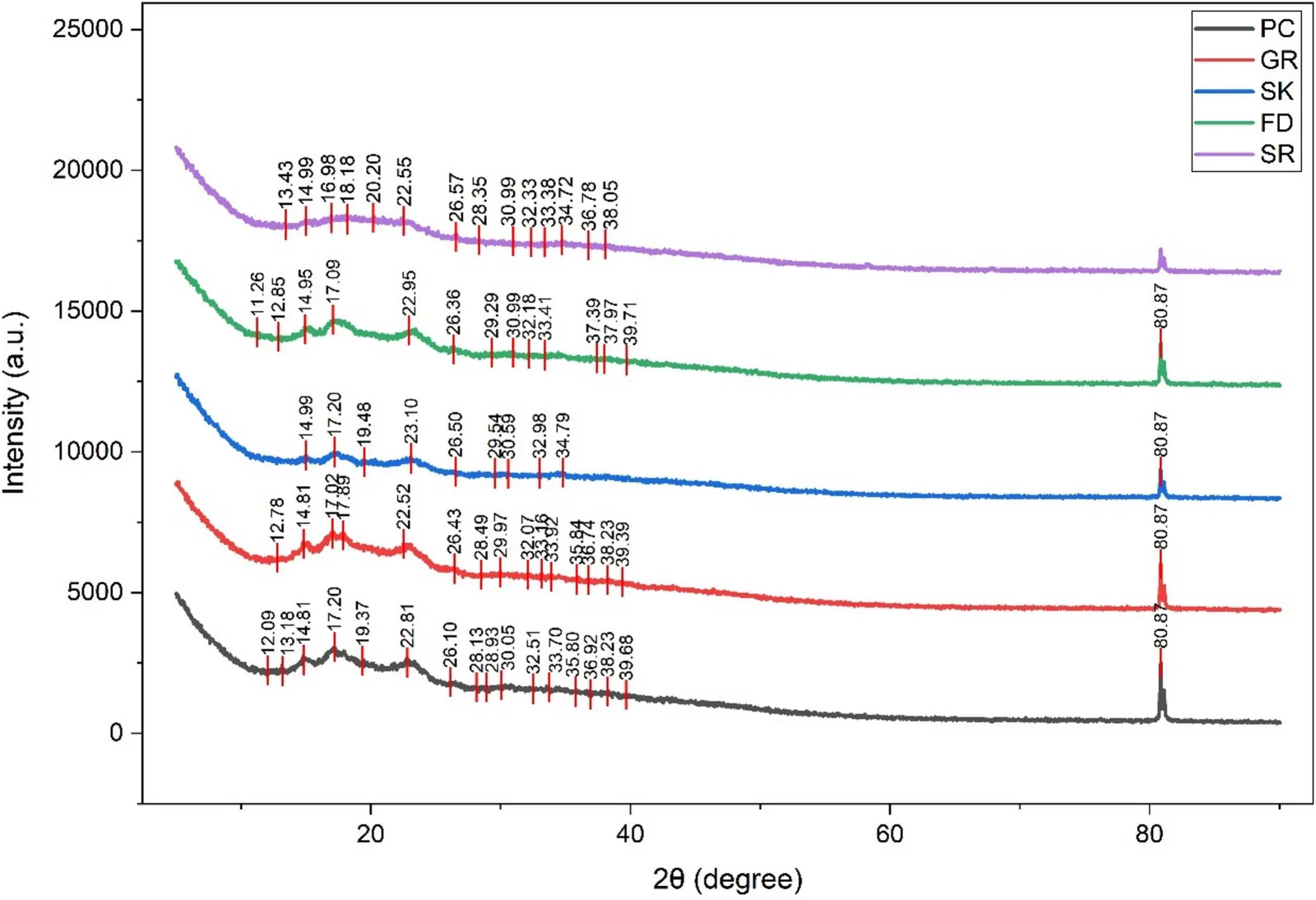
X-ray diffractograms of rice bean starch after five processing treatments: soaking (SK), germination (GR), pressure cooking (PC), sand roasting (SR), and freeze-drying (FD).

The processing treatments influenced the extent of structural ordering differently. SK largely preserved the native crystalline architecture, as hydration primarily occurs within the amorphous regions of starch granules without sufficient thermal input to disrupt the crystalline lamellae. The retention of sharp diffraction peaks indicates minimal alterations in molecular ordering and preservation of starch crystallinity (Munjal et al., 2024). Similarly, FD effectively maintains crystalline integrity by removing water through sublimation under sub-zero conditions, thereby preventing starch gelatinisation and preserving the native A-type crystalline arrangement with limited thermal disorganisation (Xia et al., 2024). GR resulted in a moderate reduction in the diffraction peak intensity, reflecting a partial loss of crystallinity. This structural modification is attributed to the activation of endogenous hydrolytic enzymes, particularly α-amylase, during sprouting, which preferentially hydrolyses amorphous starch regions, followed by the gradual degradation of crystalline domains, ultimately reducing relative crystallinity (Liu et al., 2024). Among all the treatments, PC caused the most pronounced disruption of the crystalline structure. Exposure to combined heat and moisture above the gelatinisation temperature promoted extensive swelling of starch granules and dissociation of amylopectin double helices, leading to the collapse of the ordered crystalline regions. This transformation was evidenced by the broadening and flattening of the diffraction peaks, indicating the conversion of starch from a semi-crystalline to a predominantly amorphous state (Munjal et al., 2024). SR treatment involves dry heat processing, which reduces starch crystallinity, but through thermal dextrinisation rather than hydrothermal gelatinisation. High-temperature treatment under limited moisture conditions induces cleavage and rearrangement of starch chains, resulting in altered diffraction intensity and increased structural disorder, which is distinct from moist heat-induced structural transitions (Munjal et al., 2024). A distinct sharp diffraction peak was observed at a higher 2θ angle (∼80.86°) across all processing treatments, lying beyond the conventional starch crystalline region (<30°), and was attributed to highly ordered inorganic crystalline phases. Similar XRD reflections at high 2θ angles have been reported in legume seeds and are associated with mineral complexes including calcium oxalate or phytate salts (Milán-Noris et al., 2017; Munjal et al., 2024). The persistence of this peak, despite processing-induced modifications to starch structure, highlights the coexistence of organic starch crystallinity and stable inorganic mineral phases within the rice bean matrix. XRD analysis demonstrated that FD effectively preserves native A-type starch crystallinity by preventing thermal disruption; soaking induces minimal structural changes; GR moderately reduces crystallinity through enzymatic hydrolysis; PC extensively disrupts crystalline order through gelatinisation; and SR promotes partial structural disorder through dextrinisation. The stability of mineral crystallites across treatments further confirms the resistance of inorganic components to processing-induced structural changes.

## 4 Conclusion

This study demonstrated that post-harvest processing significantly influenced the physicochemical, functional, structural, antioxidant, and metabolomic characteristics of rice beans. Among the treatments, GR was the most effective in enhancing nutritional quality, yielding the highest protein, total phenolic content, total flavonoid content, DPPH radical scavenging activity, foaming capacity, and emulsion stability. Metabolomic analysis further revealed the enrichment of bioactive flavonoids, including quercetin, kaempferol, and naringenin derivatives. FD effectively preserved bioactive metabolites, native starch crystallinity, and colour, whereas PC improved hydration-related properties, including swelling index and water absorption, making it suitable for instant food applications. Although SR reduced phenolic content and antioxidant activity, it enhanced oil absorption capacity and retained thermostable metabolites, supporting its application in lipid-rich and savoury food products. Metabolomic, FT-IR, XRD, and functional data provided complementary characterisation of processing-induced changes and demonstrated that structural modifications were closely associated with changes in the techno-functional properties of rice bean flour, providing mechanistic insights into processing-dependent quality enhancement. These findings indicate that no single processing method is universally optimal; instead, each offers distinct advantages depending on the intended food application. A limitation of this study is that all treatments were initiated from soaked beans, with no untreated raw control included. Consequently, the reported effects represent changes relative to the soaked state rather than the native seed. Future studies should incorporate raw controls and complementary metabolomic analyses using both positive and negative ionisation modes to achieve a more comprehensive characterisation of processing-induced changes. Overall, this study provides a scientific basis for the valorisation of rice bean as a sustainable, nutrient-rich ingredient and demonstrates how targeted processing strategies can be used to develop functional foods tailored to specific nutritional and technological requirements.

## Declaration of generative AI use

The authors have used generative AI during the preparation of this work to enhance the readability of the article.

## CRediT authorship contribution statement

**Somaina Basumatary:** Writing – original draft, Methodology, Investigation, Formal analysis. **Manish Kumar Singh:** Writing – original draft, Validation, Supervision, Resources, Methodology, Data curation, Conceptualization. **Baby Zaithanpuii Hmar:** Writing – review & editing, Supervision. **Arvind Kumar:** Writing – review & editing, Resources. **Jagan Karthik Sugumar:** Writing – review & editing, Methodology. **Ankur Aggarwal:** Writing – review & editing, Visualization. **Himanshu Mishra:** Writing – review & editing, Investigation. **Kumari Amita Singh:** Writing – review & editing, Resources, Methodology. **Satyender Singh Yadav:** Visualization, Software. **Sunil Kumar:** Software. **Vishal Kumar:** Writing – review & editing, Resources.

## Funding

No funding received.

## Declaration of competing interest

The authors declare that they have no known competing financial interests or personal relationships that could have appeared to influence the work reported in this paper.

## Data Availability

Data will be made available on request.
